# Association Between Metabolic and Bariatric Surgery and Melanoma Risk: A Systematic Review and Meta-analysis of Observational Studies

**DOI:** 10.1007/s11695-026-08924-7

**Published:** 2026-09-25

**Authors:** Túlio Pimentel, Maria Clara Florêncio, Mariano Gallo Ruelas, Luiz Alberto Mattos, Anna Carolina Batista Dantas, Tyler J. Loftus, Álvaro Antônio Bandeira Ferraz

**Affiliations:** 1 Federal University of Pernambuco, Recife, Brazil; 2 Faculdade Pernambucana de Saúde, Recife, Brazil; 3 Instituto de Investigación Nutricional, Lima, Peru; 4 Departamento de Cirurgia, Universidade Federal do Rio Grande do Norte, Natal, Brazil; 5 University of Florida, Gainesville, United States

**Keywords:** Bariatric surgery, Obesity, Cancer, Melanoma

## Abstract

Metabolic and bariatric surgery (MBS) has been associated with a reduced incidence of various cancers. Yet, its potential impact on melanoma, the most lethal form of skin cancer, remains unclear. This systematic review and meta-analysis aimed to explore associations between MBS and melanoma risk. We searched MEDLINE, Embase, and Cochrane for studies that compared MBS to non-MBS patients and reported the incidence of melanoma in both groups up to December 2025. The primary outcome was the risk of developing melanoma. A restricted maximum likelihood random-effects model was used to calculate risk ratios with 95% confidence intervals. Ten studies were included, encompassing 2,310,128 patients, of whom 445,947 (19.3%) underwent MBS. The overall incidence of melanoma was 0.11% in the MBS group (516 / 445,947) and 0.24% (4,498 / 1,864,181) in the control group. Slightly lower melanoma risk was observed in the MBS group, although the difference did not reach statistical significance (risk ratio 0.78; 95% CI 0.52 to 1.16; *p* = 0.222; I² = 85%). By synthesizing the largest body of evidence to date and focusing on melanoma as the primary outcome, this study provides a comprehensive assessment of the association. Considering the rarity of the outcome, the substantial heterogeneity, and the very low certainty of evidence from observational studies, further large-scale studies are warranted to better define the association between MBS and melanoma risk.

**Supplementary Information:**

The online version contains supplementary material available at https://doi.org/10.1007/s11695-026-08924-7.

## Key Points

- Obesity is a recognized risk factor for several cancers, but its association with melanoma remains uncertain and may be strongly influenced by environmental and phenotypic factors.
- This systematic review and meta-analysis synthesized 10 studies including over 2.3 million patients and found a lower absolute incidence of melanoma after MBS, but no statistically significant reduction in pooled risk.
- The certainty of evidence was very low and substantial heterogeneity was observed, underscoring the need for further high-quality, dedicated studies to clarify the association between MBS and melanoma incidence.

**Supplementary Information:**

The online version contains supplementary material available at https://doi.org/10.1007/s11695-026-08924-7.

## Introduction

Obesity has become a major public health challenge, with its prevalence more than doubling worldwide in recent decades [1]. By 2035, it is estimated that nearly 50% of adults will be overweight or have obesity, a condition associated with numerous health comorbidities, including hypertension, diabetes, and dyslipidemia [2]. Moreover, it is a well-established risk factor for various cancers, accounting for approximately 20% of all cases globally through complex mechanisms such as chronic inflammation, hormonal dysregulation, and metabolic alterations [3, 4].

Most evidence linking obesity to cancer has focused on malignancies such as esophageal, endometrial, and renal cell carcinoma [3]. Emerging data, however, suggest that melanoma, the most lethal form of skin cancer, may also be influenced by excess adiposity. Factors such as hyperinsulinemia, elevated insulin-like growth factor (IGF-1), and increased leptin levels, combined with the chronic pro-inflammatory environment associated with obesity and metabolic syndrome, have been linked to melanoma growth and proliferation [5, 6].

In this context, metabolic and bariatric surgery (MBS), in addition to promoting substantial and sustained weight loss, has been associated with lower incidence rates of several obesity-related malignancies [7, 8]. More recently, some studies have suggested that these effects might also extend to melanoma [9, 10]. However, this association remains unclear and understudied. Unlike many obesity-related cancers, melanoma is strongly influenced by environmental and phenotypic factors, particularly ultraviolet exposure and skin phototype, making residual confounding especially relevant when interpreting this association.

Evidence syntheses focused specifically on melanoma after MBS are scarce. One prior broad cancer review addressed this question and included three skin cancer cohorts, but melanoma was a subgroup outcome, and no quantitative pooling was performed [11]. A prior meta-analysis evaluating the impact of MBS on overall cancer incidence included melanoma only as a secondary outcome [7]. However, it incorporated only three studies [12–14], despite the availability of several additional eligible cohorts at the time [9, 15–17]. Furthermore, additional studies contributing data on this specific association have been published more recently [10, 18, 19]. The present systematic review and meta-analysis was conducted to address this gap by comprehensively identifying and synthesizing comparative studies of patients undergoing MBS and non-MBS control groups, with the objective of evaluating the association between MBS and the risk of developing melanoma.

## Methods

This systematic review and meta-analysis complies with the Cochrane Collaboration and the Preferred Reporting Items for Systematic Reviews and Meta-Analyses (PRISMA) statement guidelines [20] and was registered in the International Prospective Register of Systematic Reviews (PROSPERO) under protocol CRD42024620438.

### Eligibility Criteria

Studies were eligible if they were comparative randomized or non-randomized studies, including patients who underwent MBS and a control group that did not undergo MBS, and reported the incidence of melanoma in both groups. There were no restrictions on publication date, language, or type of MBS performed. Only manuscripts published as full-text were included. Studies that did not meet all inclusion criteria were excluded from the analysis. We also assessed the included studies for potentially overlapping populations, registries, databases, and study periods. When overlapping populations were suspected, study characteristics, data sources, and inclusion periods were reviewed to avoid duplicate inclusion of the same patient population.

### Search Strategy and Data Extraction

We conducted a comprehensive systematic search of MEDLINE, Embase, and the Cochrane Central Register of Controlled Trials (CENTRAL) from inception to December 5, 2025. The search strategy included the following terms: (“bariatric surgery” OR “gastric sleeve” OR “gastric bypass” OR “weight loss surgery” OR “metabolic surgery” OR “obesity surgery” OR “weight reduction surgery”) AND (“skin cancer” OR “cancer” OR “melanoma”). In addition, we manually reviewed the references of all included studies and relevant systematic reviews to identify additional eligible studies. Duplicate records were manually identified and removed using the Rayyan Platform, a web-based tool for systematic reviews. Two authors (TP and MCF) independently assessed the eligibility of studies by screening titles and abstracts, followed by independent full-text review of potentially eligible studies. Any disagreements were resolved by consensus between the two reviewers.

The same two authors independently extracted data from eligible studies using a predefined Excel sheet: first author, year, country, study design and conduct, mean age, mean body mass index (BMI), type of MBS, and the incidence of melanoma in MBS and control groups. Disagreements about data accuracy were resolved via consultation with the other authors.

### Outcomes

Our primary outcome was the relative risk of developing melanoma among subjects who undergo MBS.

### Quality Assessment

Two independent reviewers (TP and MGR) assessed the risk of bias in each study using the Risk of Bias in Non-randomized Studies of Interventions Tool (ROBINS-I) Version 2 (https://www.riskofbias.info/welcome/robins-i-v2*)* [21]. The tool comprises seven domains: (1) risk of bias due to confounding, (2) risk of bias in classification of interventions, (3) risk of bias in selection of participants into the study (or into the analysis), (4) risk of bias due to deviations from intended interventions, (5) risk of bias due to missing data, (6) risk of bias arising from measurement of the outcome, and (7) risk of bias in selection of the reported results. Studies were included if they were classified as low or moderate risk in most domains. This criterion referred to domain-level judgments rather than the overall risk-of-bias rating.

### Certainty of Evidence

The Grading of Recommendations Assessment, Development, and Evaluation (GRADE) framework was applied to evaluate the overall certainty of the evidence (CoE) [22]. The CoE could be downgraded if concerns were identified in any of the following domains: (1) risk of bias; (2) inconsistency; (3) indirectness; (4) imprecision; and (5) publication bias. Conversely, in the absence of major concerns, the CoE was upgraded in cases where a substantial effect size was observed. The final classification of CoE was categorized as very low, low, moderate, or high.

### Statistical Analysis

Risk ratios (RRs) with 95% confidence intervals (CIs) were calculated for binary outcomes. When raw melanoma event counts were not directly reported, approximate event numbers were derived from the reported incidence rates and the group-specific mean follow-up duration. Heterogeneity was assessed using Cochran’s Q test and I² statistics, with p-values less than 0.10 and I² values greater than 50% indicating significant heterogeneity. Pooled risk ratios were estimated using a random-effects model for binary outcomes, with between-study variance estimated by restricted maximum likelihood. Analyses were conducted in R version 4.4.2 using the meta package and the metabin() function. For studies with zero melanoma events in one comparison group, risk ratios were estimated using the default continuity correction implemented in the meta package. For studies reporting data as medians with interquartile ranges or medians with ranges, values were converted to means and standard deviations (SD) using the methods outlined by Wan et al. [23] and Luo et al. [24]. A leave-one-out sensitivity analysis was performed by sequentially omitting one study at a time to assess the robustness of the results. A sensitivity analysis excluding studies judged as having serious or critical overall risk of bias was also performed to assess the robustness of the pooled estimate. Subgroup analyses by follow-up duration, study region, regional latitude, sample size, and type of surgery were prespecified in the study protocol; however, these were not conducted because the available data did not allow for methodologically robust or reliable evaluations.

## Results

### Study Selection and Characteristics

**Fig. 1:**
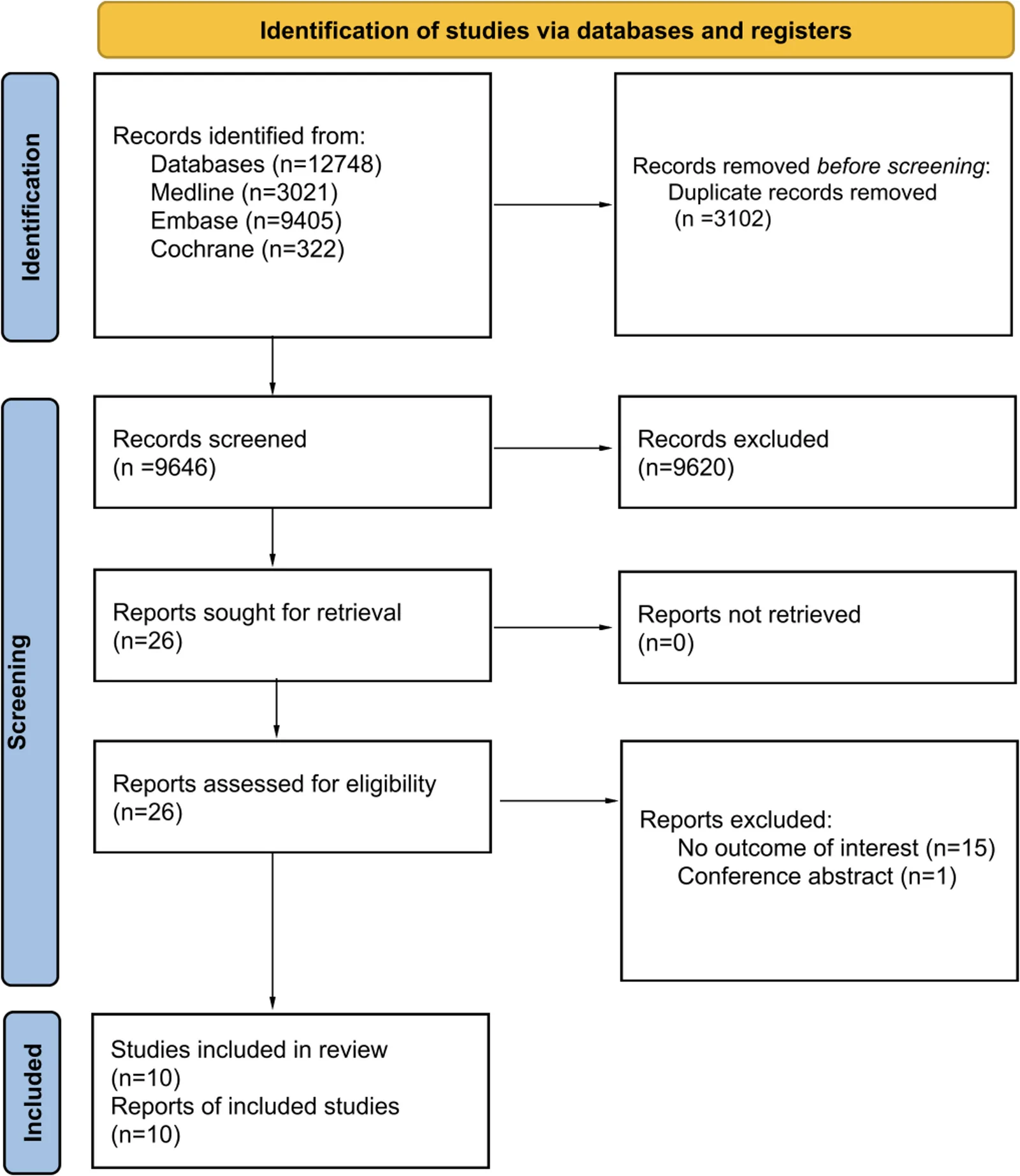
PRISMA flow diagram of study screening and selection

The initial search across three electronic databases yielded 12,748 articles. After removing 3,102 duplicates, the remaining 9,646 articles were screened based on their titles and abstracts, resulting in 26 studies selected for full-text review. Among them, 16 studies did not meet the inclusion criteria and were excluded (Supplementary Table S1). Ultimately, ten studies were included in the systematic review and meta-analysis (Fig. 1).

The included studies [9, 10, 12–19] encompassed a total of 2,310,128 patients, of whom 445,947 (19.3%) underwent MBS. The study designs consisted of nine retrospective studies and one prospective study [9]. The publication dates ranged from 2008 to 2025, with a mean follow-up period varying between 5 and 17.9 years. The mean age of participants ranged from 38.9 to 51.8 years, and their mean BMI ranged from 40.1 to 51.6 kg/m^2^. The types of MBS assessed across the studies included roux-en-Y gastric bypass (RYGB), sleeve gastrectomy (SG), vertical banded gastroplasty (VBG), and adjustable gastric banding (AGB). Geographically, seven studies [12, 14–19] were conducted in North America, while three [9, 10, 13] originated from Europe. Detailed characteristics of the included studies are presented in Table 1.

**Table 1:** Characteristics of included studies

Study	Design	Time Period	Database	Type of MBS	Mean Follow-up (years)	Total Sample	Number of Patients MBS/C	Mean Age (years) MBS/C	Female (%) MBS/C	Mean BMI (kg/m^2^) MBS/C	Melanoma Events (%) MBS/C
Adams et al. [12]	Retrospective	1984–2002	Utah Cancer Registry (USA)	RYGB	12.5	16,038	6596/9442	38.9/39.1	86/83	44.9/47.4	0.25/0.3
Aminian et al. [15]	Retrospective	2004–2017	Cleveland Clinic Health System (USA)	RYGB (66%) SG (34%)	6.2	30,318	5053/25,265	46/45.6	76.9/77.2	46/45.3	0.27/0.12
Christou et al. [16]	Retrospective	1986–2002	McGill University Health Centre, RAMQ database (Canada)	RYGB (79.2%) VBG(18.7%) LRYGB (2.1%)	5	6781	1035/5746	45.1/46.7	65.6/64	NA	0.19/0.47
Dongur et al. [18]	Retrospective	2000–2023	TriNetX database (USA)	RYGB (73.9%) SG (26.1%)	5	306	153/153	49.4/50.3	67.3/66	42.9/42.5	0.65/1.96
Lazzati et al. [13]	Retrospective	2010–2017	PMSI database (France)	Open or laparoscopic AGB, SG, and RYGB	6.3	1,140,347	288,604/851,743	39.8/51.8	81.6/55.2	NA	0.03/0.07
McCawley et al. [17]	Retrospective	1990–2006	University of Virginia Surgery Department Database (USA)	LRYGB (61.7%) RYGB (27.3%) Others (11%)	NA	4977	1482/3495	41.7/46.9	100/100	51.6/NA	0.13/0
Miller et al. [19]	Retrospective	2001–2019	Single integrated multispecialty group Midwestern Health System (USA)	LRYGB LSG	6.6	3749	1593/2156	46.2/46.5	79/78.6	46.2/45.2	0.31/0.46
Stenberg et al. [10]	Retrospective	2007–2020	Scandinavian Obesity Surgery Registry	RYGB LSG	7.8	709,368	68,424/640,944	40.5/40.1	75.7/74.7	41.8/NA	0.4/0.52
Taube et al. [9]	Prospective	1987–2001	Swedish Obese Subjects (SOS) Study (Sweden)	VBG (68.1%) AGB (18.7%) RYGB (13.2%)	17.9	4047	2007/2040	47.2/48.7	70.8/70.9	42.4/40.1	0.59/1.42
Tsui et al. [14]	Retrospective	2006–2012	The New York Statewide Planning and Research Cooperative System database (USA)	LSG LRYGB LAGB	NA	394,197	71,000/323,197	44/45	78.5/76.4	NA	0.14/0.12

*AGB* adjustable gastric banding, *MBS* metabolic and bariatric surgery, *C* control, *LAGB* laparoscopic adjusted gastric banding, *RYGB* roux-en-Y gastric bypass, *LRYGB* laparoscopic roux-en-Y gastric bypass, *LSG* laparoscopic sleeve gastrectomy, *SG* sleeve gastrectomy, *VBG* vertical banded gastroplasty, *NA* not available

### Melanoma

**Fig. 2:**
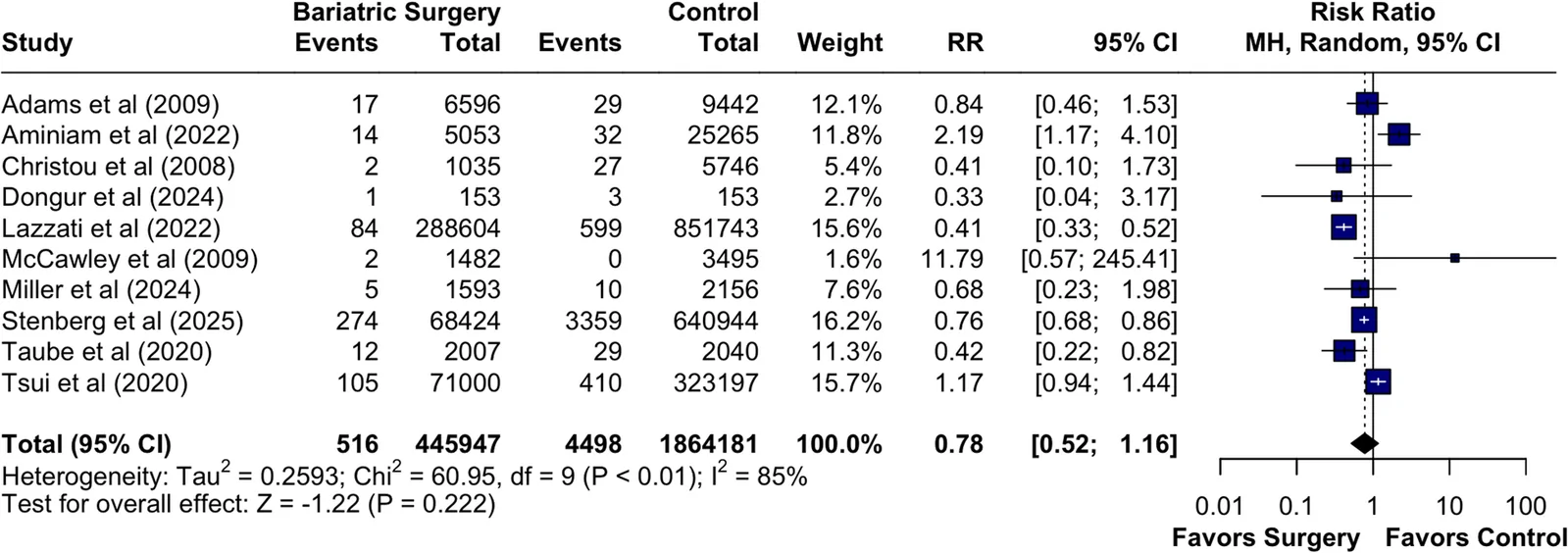
Forest plot of the association between metabolic and bariatric surgery (MBS) and melanoma risk, using risk ratios (RRs) pooled with a random-effects model. The pooled estimate showed no statistically significant association

**Fig. 3:**
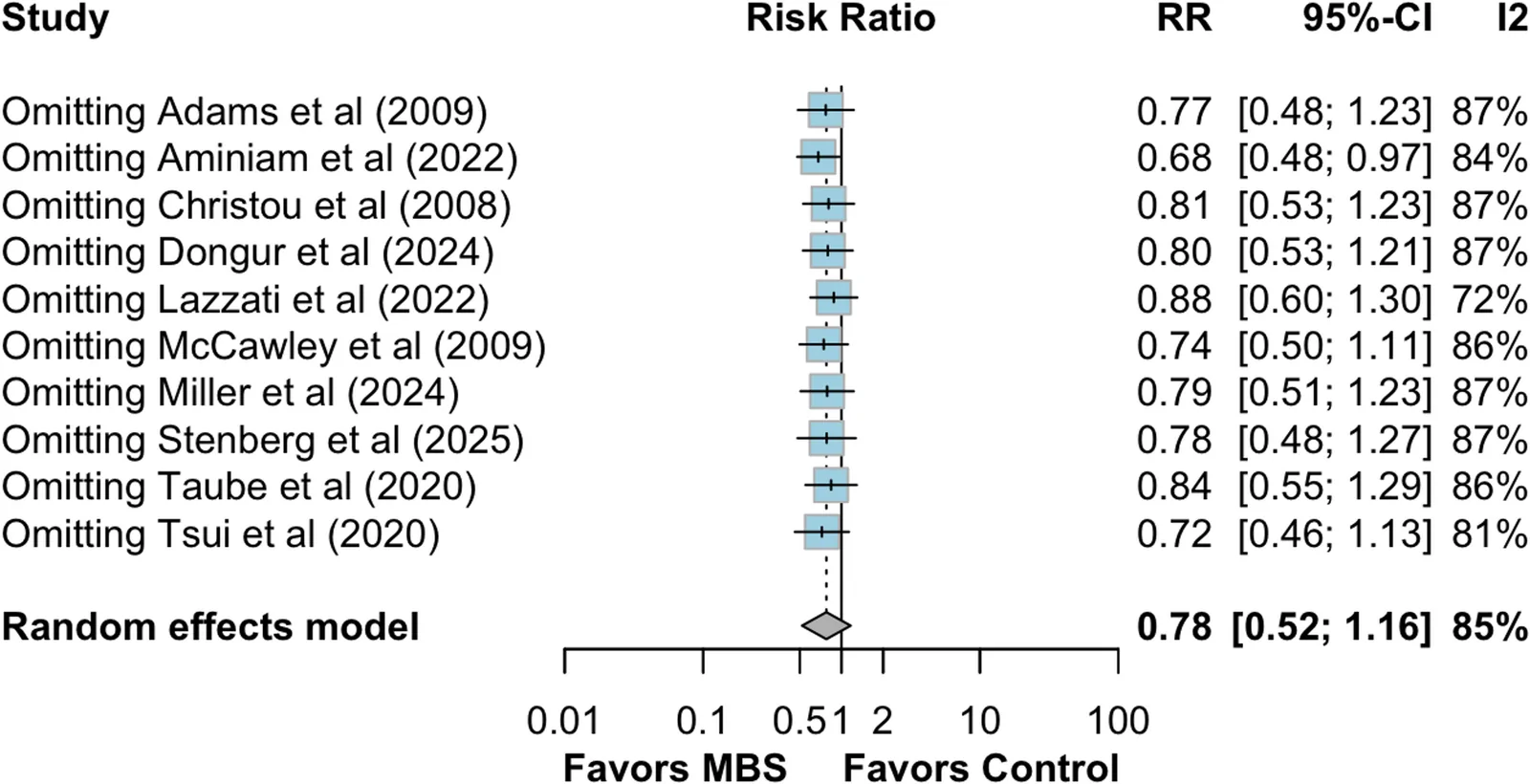
Leave-one-out sensitivity analysis for melanoma risk. Sequential exclusion of individual studies did not meaningfully resolve heterogeneity. Exclusion of Aminiam et al. yielded a statistically significant reduction in melanoma risk

**Fig. 4:**
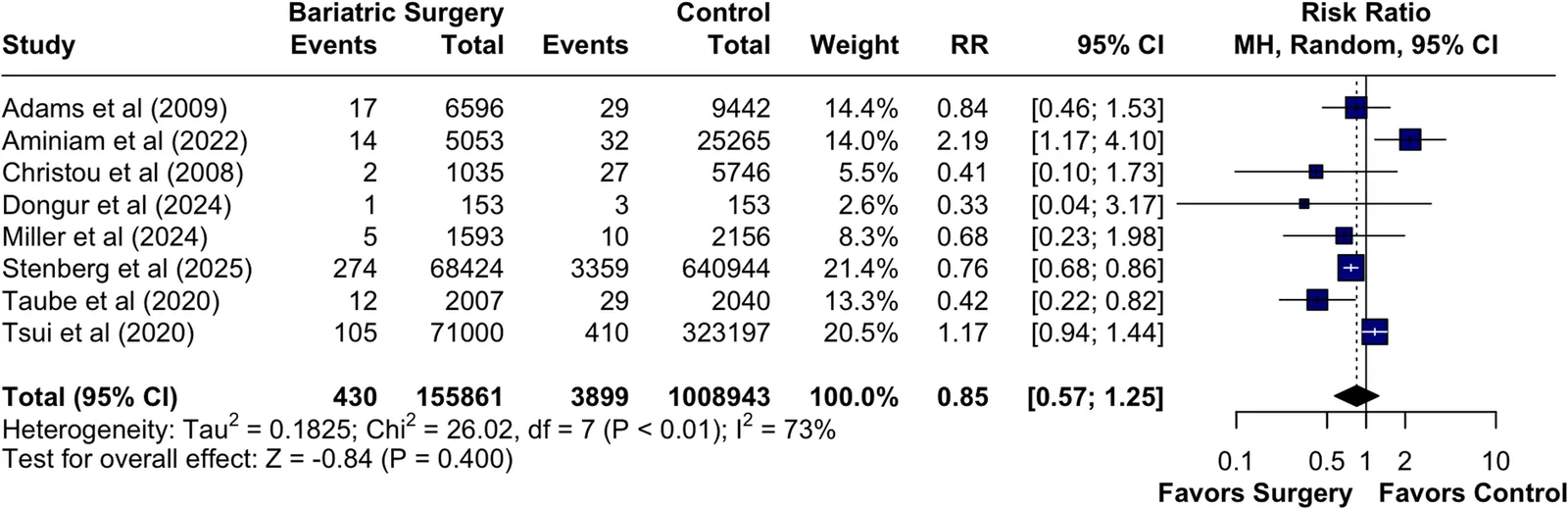
Sensitivity analysis excluding studies judged as having serious or critical overall risk of bias. The pooled estimate was not statistically significant, and substantial heterogeneity remained

The overall incidence of melanoma was 0.11% in the MBS group (516/445,947) and 0.24% in the control group (4,498/1,864,181). One study reported incidence rates per 100,000 person-years rather than raw counts [10]. For this study, we derived approximate event numbers from the reported rates and the group-specific mean follow-up. Pooled data from the 10 included studies revealed no statistically significant difference in the risk of developing melanoma between groups, with high heterogeneity (RR 0.78; 95% CI 0.52 to 1.16; *p* = 0.222; I² = 85%; Tau² = 0.25; Fig. 2). In the leave-one-out sensitivity analysis, omitting Aminian et al. [15] led to a statistically significant result, without a meaningful reduction in heterogeneity (RR 0.68; 95% CI 0.48 to 0.97; I² = 84%; Fig. 3). A sensitivity analysis excluding studies judged as having serious or critical overall risk of bias showed no material change in the pooled estimate or heterogeneity (RR 0.85; 95% CI 0.57 to 1.25; I² = 73%; Fig. 4).

### Quality Assessment

In the risk of bias assessment, seven studies [9, 10, 12, 14, 16, 18, 19] were classified as having a moderate risk of bias. One study [15] was classified as having a low risk of bias, except for concerns regarding residual confounding. Another study [13] was deemed to have a serious risk of bias due to bias in the selection of participants for the analysis and missing data. Additionally, one study [17] was considered to have a critical risk of bias due to confounding, as it did not employ methods to control for potential confounders. The results of the risk of bias assessment are presented in Table 2.

**Table 2:** ROBINS-I risk of bias assessment

Domain	Adams et al. [12]	Aminian et al. [15]	Christou et al. [16]	Dongur et al. [18]	Lazzati et al. [13]	McCawley et al. [17]	Miller et al. [19]	Stenberg et al. [10]	Taube et al. [9]	Tsui et al. [14]
Bias due to confounding	Moderate	Low, except for concerns with residual confounding	Moderate	Moderate	Low, except for concerns with residual confounding	Critical	Moderate	Low, except for concerns with residual confounding	Low, except for concerns with residual confounding	Moderate
*Bias in classification of interventions*	Low	Low	Low	Low	Low	Low	Low	Low	Low	Low
Bias in selection of participants into the analysis	Low	Low	Low	Low	Serious	Moderate	Low	Low	Low	Low
Bias due to deviations from intended interventions	Low	Low	Low	Low	Low	Low	Low	Low	Low	Low
Bias due to missing data	Low	Low	Low	Low	Serious	Low	Low	Low	Low	Low
Bias in measurement of the outcome	Low	Low	Moderate	Moderate	Moderate	Moderate	Moderate	Moderate	Moderate	Moderate
Bias in selection of the reported result	Low	Low	Low	Low	Low	Low	Low	Low	Low	Low
Overall	Moderate	Low, except for concerns with residual confounding	Moderate	Moderate	Serious	Critical	Moderate	Moderate	Moderate	Moderate

### Grading of Recommendations Assessment, Development and Evaluation

The GRADE assessment determined that the certainty of the evidence (CoE) for overall melanoma incidence was *very low*. The main reasons for downgrading the evidence included concerns related to confounding, selection bias, and inconsistent between-groups outcome measurement (Table 3).

**Table 3:** Summary of findings (SoF) of GRADE assessment

Summary of Findings for MBS Versus No MBS for prevention of melanoma							
Outcome and follow-up	Patients (studies), N	Relative effect (95% CI)	**Absolute effects (95% CI)**			Certainty	What happens
			**No MBS**	**MBS**	**Difference**		
Overall melanoma incidence	2,310,128 (10 non-randomized studies)	**RR = 0.78** (0.52 to 1.16)	24 per 10.000	**12 per 10.000**	**12 fewer per 10.000** (from 7 fewer to 14 more)	⨁◯◯◯ Very low^a, b,c^	The overall evidence is very uncertain about the effect of MBS on melanoma incidence.

*CI* confidence interval, *RR* risk ratio, *MBS* Metabolic and Bariatric Surgery

a. Risk of bias: Downgraded two levels due to studies having concerns with residual confounding, selection bias, and bias in the outcome measurement

b. Inconsistency: Downgraded one level due to different point estimates in three studies, accounting for 30% of the meta-analysis weight

c. Imprecision: Pooled effect estimate includes possible benefits and harm; no definitive conclusions can be drawn

## Discussion

In this systematic review and meta-analysis of 10 studies involving a total of 2,310,128 patients, melanoma was less frequently observed after MBS (0.11% vs. 0.24%), although the reduction in risk was not statistically significant. However, the very low CoE, high heterogeneity, and rarity of the outcome suggest that these findings should be interpreted with caution.

Obesity is a recognized risk factor for several cancers, driven by metabolic and inflammatory mechanisms. Key pathways include hyperinsulinemia, elevated IGF-1, and chronic inflammation involving cytokines like tumor necrosis factor-alpha (TNF-α) and interleukins [3, 25]. These factors, along with oxidative stress, create a pro-tumorigenic environment that promotes DNA damage and tumor growth [4, 26]. In line with this, MBS has been associated with a significant reduction in the incidence of obesity-related cancers, potentially by improving metabolic regulation, decreasing systemic inflammation, and reducing the expression of cancer-related biomarkers, such as apolipoprotein B and thrombospondin-2, with these effects likely attributed to the weight loss achieved after surgery [27, 28]. Melanoma, the most lethal form of skin cancer, is increasing at a faster rate than any other malignancy, particularly among fair-skinned populations [29]. It arises from a complex interplay of genetic predispositions and environmental exposures, and despite growing awareness, the connection between melanoma and obesity remains incompletely understood [30].

Evidence suggests a possible link between obesity-related metabolic dysfunction and melanoma development, driven by several potential mechanisms. Leptin, a hormone secreted by adipocytes, has been associated with increased melanoma growth and metastasis [31, 32]. Melanoma cells express receptors for both insulin and IGF-1, signaling pathways that are overactivated in obesity and may directly contribute to melanoma proliferation [9]. Additionally, adipocytes in the tumor microenvironment can act as metabolic support for melanoma cells, providing a rich source of energy substrates, such as free fatty acids, which fuel tumor growth and metabolic reprogramming [33]. Meta-analyses have identified a 25% to 31% increased risk of melanoma among men with obesity [8, 34]. These findings underscore the need for a deeper understanding of the association between weight-loss interventions, such as MBS, and melanoma risk.

Beyond melanoma incidence, obesity may also influence tumor aggressiveness at presentation. Studies have reported an association between higher BMI and greater Breslow thickness at the time of melanoma diagnosis [35, 36]. Importantly, severe obesity (BMI > 35 kg/m²) has also been associated with an approximately twofold higher likelihood of lymph node metastasis [36]. Although the studies included in the present review did not directly investigate the impact of MBS on melanoma spread, invasiveness, or metastatic behavior, these observations suggest that obesity-related metabolic and inflammatory disturbances may contribute not only to melanoma development but also to a more aggressive disease phenotype.

Paradoxically, obesity has also been associated with improved outcomes in patients with advanced melanoma treated with immune checkpoint inhibitors, a phenomenon often referred to as the “obesity paradox” [37, 38]. One proposed mechanism is that obesity may promote immune dysregulation and increased PD-1 expression, thereby enhancing responsiveness to PD-1 blockade in some settings [37]. More recently, studies incorporating body composition analysis have suggested that this relationship is more complex than BMI alone, with greater skeletal muscle mass being associated with improved survival, whereas excess visceral adiposity, more closely linked to systemic inflammation, has been associated with worse outcomes, possibly through impairment of antitumor immune responses [39, 40]. Together, these findings highlight the complex interplay between obesity, body composition, melanoma progression, and immunotherapy response, and that the potential impact of MBS on these outcomes remains to be better elucidated.

The impact of postoperative weight loss magnitude on melanoma incidence remains uncertain. Across the included studies that reported weight-loss outcomes, the available findings were heterogeneous and did not suggest a consistent dose–response relationship. Aminian et al. [15] reported a mean weight loss of 27.5% in the bariatric group, yet melanoma incidence was numerically higher in the surgical cohort than in controls (0.27% vs. 0.12%). In contrast, Christou et al. [16] reported 62.1% excess weight loss, accompanied by a lower melanoma incidence in the bariatric group (0.19% vs. 0.47%), while Taube et al. [9] reported a mean weight loss of 21.6 kg at 15-year follow-up, also with a lower melanoma incidence after surgery (0.59% vs. 1.42%). Although these comparisons should be interpreted with caution given differences in study design, follow-up, and weight-loss reporting, they suggest that any potential association between MBS and melanoma risk may not depend solely on the degree of weight loss, but also on broader metabolic, hormonal, and inflammatory changes induced by surgery.

Our results are consistent with a previous meta-analysis on cancer incidence, which found no significant reduction in subgroup analyses of melanoma risk after MBS, although a lower overall incidence of melanoma was noted. Chen et al. [7] pooled three studies and reported no significant difference in melanoma risk between MBS and control groups. The present review synthesized evidence from 10 studies and specifically focused on melanoma as the primary outcome, thereby providing a more comprehensive and detailed evaluation of this association. In addition, the sensitivity analysis showed that exclusion of Aminian et al. [15] resulted in a statistically significant reduction in melanoma risk, although heterogeneity persisted, supporting the uncertainty and the need for cautious interpretation of the overall evidence.

It is important to highlight the limited evidence available on this association. Among the included studies, only Taube et al. [9] conducted a prospective analysis specifically evaluating melanoma as a main outcome, whereas most others assessed overall cancer incidence after MBS, with melanoma reported only in subgroup analyses. In a Swedish cohort of 4,047 patients followed for a mean of 17.9 years, Taube et al. [9] demonstrated that MBS was associated with nearly a 60% reduction in melanoma risk compared with contemporaneously matched controls (adjusted subhazard ratio: 0.43; 95% CI: 0.21–0.87; *P* = 0.02). Similarly, Stenberg et al. [10], in another large Swedish nationwide cohort including 68,424 patients undergoing MBS and 640,944 matched controls, also found a significant reduction in melanoma incidence after surgery (incidence rate ratio: 0.77; 95% CI: 0.68–0.87).

The consistent findings from Swedish cohorts also raise the possibility that skin phototype may modulate this association. Populations in Sweden are predominantly fair-skinned, with a higher baseline susceptibility to UV-induced melanoma [41]. In this context, the protective effect of MBS against melanoma might be more easily detected in a high-risk population. Beyond skin phototype, additional region-specific factors—such as genetic background, cultural patterns of sun exposure, latitude-related UV intensity, and lifestyle behaviors—may also contribute to these observations. Clarifying whether similar associations are observed across populations with different demographic, genetic, and environmental characteristicsremains an open question requiring further investigation in more diverse cohorts.

Taken together, current evidence suggests a biologically plausible link between obesity, weight loss after MBS, and melanoma risk, yet the scarcity of dedicated studies, conflicting findings, and heterogeneity across cohorts limit firm conclusions. While the Swedish data point toward a potential protective effect, the generalizability of these results remains uncertain. Further high-quality, prospective studies across diverse populations are needed to clarify the association of MBS with melanoma incidence and to better define the mechanisms underlying this association.

Although our pooled analysis was based on crude melanoma event counts, adjusted melanoma-specific estimates were reported in a limited number of studies. Taube et al. [9] reported an adjusted subhazard ratio showing a significant reduction in melanoma risk after MBS (adjusted subhazard ratio: 0.43; 95% CI: 0.21–0.87; *P* = 0.02). In contrast, Adams et al. [12] reported no statistically significant association between MBS and melanoma risk in an adjusted analysis (HR: 0.71; 95% CI: 0.38–1.34; *P* = 0.30). Therefore, a pooled analysis of adjusted estimates was not feasible, and this should be considered when interpreting the crude event-based pooled results.

### Implications for Practice, Policy, and Future Research

The present findings suggest that, although MBS may reduce the incidence of several obesity-related cancers, its association with melanoma remains uncertain. For clinical practice, this underscores that melanoma should not currently be considered a primary driver in surgical decision-making for obesity treatment. At the policy level, these results highlight the need to strengthen cancer surveillance in bariatric cohorts and to ensure that melanoma is systematically assessed in future registries and outcome studies. Future research should focus on large, prospective, and ethnically diverse cohorts specifically designed to evaluate melanoma as a primary outcome, as well as mechanistic studies exploring how metabolic and inflammatory changes after MBS might interact with melanoma biology.

### Strengths and Limitations

To our knowledge, this is the first systematic review and meta-analysis specifically dedicated to analyzing the incidence of melanoma after bariatric surgery and the largest synthesis to date on this topic. Previous research [7, 11] relied on data from only three studies and considered melanoma only as part of subgroup analyses. In contrast, our study offers a comprehensive evaluation of melanoma risk following bariatric surgery, incorporating all available evidence to investigate how bariatric surgery might be associated melanoma risk. However, the large overall sample size should not be interpreted as indicating high certainty of evidence, as melanoma events were rare and the certainty of evidence was very low.

Among the included studies, only one was prospective and primarily focused on melanoma incidence, while the others were retrospective and analyzed melanoma as part of subgroup analyses. Additionally, there was considerable heterogeneity in the periods of patient inclusion, follow-up durations (ranging from 5 to 17.9 years), and the types of MBS performed. These differences may limit the comparability of cumulative incidence estimates, as studies with longer follow-up periods may detect more incident melanoma cases than studies with shorter observation windows.

Our analysis also has limitations. First, the small number of included studies reduces the precision of our findings and limits the depth of our conclusions. This limited data also restricted a formal assessment of publication bias, leaving the potential influence of unpublished studies uncertain. Despite conducting leave-one-out sensitivity analyses, the substantial heterogeneity observed across studies could not be fully resolved, which may have affected the consistency and interpretability of our results. In addition, because included studies were observational, the findings may be affected by confounding by indication, as patients undergoing MBS may differ systematically from controls in baseline health status, healthcare utilization, socioeconomic status, and cancer surveillance. Detection bias is also possible, since patients undergoing MBS may have more frequent postoperative medical contact, increasing opportunities for melanoma detection compared with non-surgical controls. Furthermore, immortal time bias, differences in index-date alignment, and competing risk of death during long-term follow-up may have influenced the observed cumulative incidence estimates. Finally, other important determinants, such as lifestyle habits and skin phenotype, which are known to influence melanoma risk, could not be analyzed due to limited data from included studies.

## Conclusion

In this systematic review and meta-analysis, MBS was associated with a lower absolute incidence of melanoma; however, the pooled reduction in risk did not reach statistical significance. Given the very low certainty of evidence, high heterogeneity, rarity of melanoma events, and observational nature of the included studies, causal inference is not possible. While two large Swedish cohorts reported a significant reduction in melanoma risk after MBS, these findings may reflect population-specific factors and are not directly generalizable. Overall, the available evidence highlights the need for high-quality, prospective studies including both diverse patient populations and well-characterized subgroups to clarify whether the association between bariatric surgery and melanoma incidence varies across specific populations.

## Supplementary Information

Below is the link to the electronic supplementary material.

Supplementary Material 1 (DOCX 16.9 KB)

## Data Availability

The data extracted and analyzed during the present systematic review and meta-analysis are available from the corresponding author upon reasonable request.
