# Supplementary material for: Association Between Metabolic and Bariatric Surgery and Melanoma Risk: A Systematic Review and Meta-analysis of Observational Studies
Source: Obes Surg. 2026 Sep 25;36(11):5921–32. doi: 10.1007/s11695-026-08924-7 (PMC13714068; doi:10.1007/s11695-026-08924-7)
Supplement: Supplementary file 1 [file 11695_2026_8924_MOESM1_ESM.docx]

**Supplementary Material**

**Association Between Metabolic and Bariatric Surgery and Melanoma Risk: A Systematic Review and Meta-analysis of Observational Studies**

| 1 | Wei Y, Wong SKH, Wu T, Law BTT, Ng EKW, Lam CLK, Wong CKH. Association between bariatric surgery and risks of cancer among Chinese patients with type 2 diabetes mellitus: A retrospective cohort study. J Diabetes. 2021 Nov;13(11):868-881. doi: 10.1111/1753-0407.13179. Epub 2021 Apr 14. PMID: 33728788. | Melanoma was not reported |
| --- | --- | --- |
| 2 | Gerber P, Naqqar D, von Euler-Chelpin M, Kauppila JH, Santoni G, Holmberg D. Incidence of Cancer and Cardiovascular Disease After Bariatric Surgery in Older Patients. JAMA Netw Open. 2024 Aug 1;7(8):e2427457. doi: 10.1001/jamanetworkopen.2024.27457. PMID: 39136945; PMCID: PMC11322843. | Melanoma was not reported |
| 3 | Chittajallu V, Mansoor E, Perez J, Omar YA, Firkins SA, Yoo H, Baggott B, Simons-Linares R. Association of Bariatric Surgery with Risk of Incident Obesity-Associated Malignancies: a Multi-center Population-Based Study. Obes Surg. 2023 Dec;33(12):4065-4069. doi: 10.1007/s11695-023-06926-3. Epub 2023 Nov 16. PMID: 37971573. | Melanoma was not reported |
| 4 | Sjöholm K, Andersson-Assarsson JC, Kristensson FM, Hjorth S, Garelius HG, Jacobson P, Svensson PA, Ahlin S, Carlsson B, Peltonen M, Carlsson LMS, Taube M. Long-term incidence of haematological cancer after bariatric surgery or usual care in the Swedish Obese Subjects study: a prospective cohort study. Lancet Healthy Longev. 2023 Oct;4(10):e544-e551. doi: 10.1016/S2666-7568(23)00141-1. Epub 2023 Sep 13. PMID: 37716360. | Melanoma was not reported |
| 5 | Adams TD, Meeks H, Fraser A, Davidson LE, Holmen J, Newman M, Ibele AR, Playdon M, Hardikar S, Richards N, Hunt SC, Kim J. Long-term cancer outcomes after bariatric surgery. Obesity (Silver Spring). 2023 Sep;31(9):2386-2397. doi: 10.1002/oby.23812. PMID: 37605634; PMCID: PMC10449359. | Melanoma was not reported |
| 6 | Khalid SI, Maasarani S, Wiegmann J, Wiegmann AL, Becerra AZ, Omotosho P, Torquati A. Association of Bariatric Surgery and Risk of Cancer in Patients With Morbid Obesity. Ann Surg. 2022 Jan 1;275(1):1-6. doi: 10.1097/SLA.0000000000005035. PMID: 34183506. | Melanoma was not reported |
| 7 | Tranchart H, Gaillard M, Kassir R, Gronnier C, Moszkowicz D, Ben Amor I, Lainas P, Savala N, Meunier H, Le Roux Y, Degrandi O, Baratte C, Vychnevskaia K, Gugenheim J, Dagher I. Malignancy in bariatric surgery patients: a French multisite cohort. Surg Endosc. 2021 Nov;35(11):6021-6030. doi: 10.1007/s00464-020-08091-5. Epub 2020 Oct 19. PMID: 33078225. | Melanoma was not reported |
| 8 | Kao YK, Lin HY, Chen CI, Su YC, Chen JH. The Bariatric Surgery Is Associated with a Lower Incidence of Malignancy: Real World Data from Taiwan. Obes Surg. 2021 Sep;31(9):4015-4023. doi: 10.1007/s11695-021-05511-w. Epub 2021 Jun 5. PMID: 34089441. | Melanoma was not reported |
| 9 | Doumouras AG, Hong D, Lee Y, Tarride JE, Paterson JM, Anvari M. Association Between Bariatric Surgery and All-Cause Mortality: A Population-Based Matched Cohort Study in a Universal Health Care System. Ann Intern Med. 2020 Nov 3;173(9):694-703. doi: 10.7326/M19-3925. Epub 2020 Aug 18. Erratum in: Ann Intern Med. 2021 May;174(5):735. doi: 10.7326/L20-1240. PMID: 32805135. | Melanoma was not reported |
| 10 | Gómez CO, Romero-Funes D, Aleman R, Frieder JS, Henrique J, Gutierrez-Blanco D, White KP, Lo Menzo E, Szomstein S, Rosenthal RJ. Bariatric surgery decreases the number of first-time hospital admissions for cancer in severely obese patients. A retrospective analysis of the National Inpatient Sample database. Surg Obes Relat Dis. 2020 Nov;16(11):1648-1654. doi: 10.1016/j.soard.2020.07.017. Epub 2020 Jul 28. PMID: 32847762. | Melanoma was not reported |
| 11 | Rustgi VK, Li Y, Gupta K, Minacapelli CD, Bhurwal A, Catalano C, Elsaid MI. Bariatric Surgery Reduces Cancer Risk in Adults With Nonalcoholic Fatty Liver Disease and Severe Obesity. Gastroenterology. 2021 Jul;161(1):171-184.e10. doi: 10.1053/j.gastro.2021.03.021. Epub 2021 Mar 18. PMID: 33744305. | Melanoma was not reported |
| 12 | Tao W, Santoni G, von Euler-Chelpin M, Ljung R, Lynge E, Pukkala E, Ness-Jensen E, Romundstad P, Tryggvadottir L, Lagergren J. Cancer Risk After Bariatric Surgery in a Cohort Study from the Five Nordic Countries. Obes Surg. 2020 Oct;30(10):3761-3767. doi: 10.1007/s11695-020-04751-6. Epub 2020 Jun 13. PMID: 32535785; PMCID: PMC7467909. | Melanoma was not reported |
| 13 | Schauer DP, Feigelson HS, Koebnick C, Caan B, Weinmann S, Leonard AC, Powers JD, Yenumula PR, Arterburn DE. Bariatric Surgery and the Risk of Cancer in a Large Multisite Cohort. Ann Surg. 2019 Jan;269(1):95-101. doi: 10.1097/SLA.0000000000002525. PMID: 28938270; PMCID: PMC6201282. | Melanoma was not reported |
| 14 | Mackenzie H, Markar SR, Askari A, Faiz O, Hull M, Purkayastha S, Møller H, Lagergren J. Obesity surgery and risk of cancer. Br J Surg. 2018 Nov;105(12):1650-1657. doi: 10.1002/bjs.10914. Epub 2018 Jul 13. PMID: 30003539. | Melanoma was not reported |
| 15 | Aravani A, Downing A, Thomas JD, Lagergren J, Morris EJA, Hull MA. Obesity surgery and risk of colorectal and other obesity-related cancers: An English population-based cohort study. Cancer Epidemiol. 2018 Apr;53:99-104. doi: 10.1016/j.canep.2018.01.002. Epub 2018 Feb 3. PMID: 29414638; PMCID: PMC5865073. | Melanoma was not reported |
| 16 | 261 Examining the risk of new skin cancers among obese patients who undergo bariatric surgery: A multicenter analysis Raiker, R. et al.Journal of Investigative Dermatology, Volume 142, Issue 8, S44 | Conference Abstract |

**Supplementary Table S1** List of excluded studies with reasons
